# Epidemiological and phylogenetic characterization of human respiratory syncytial virus associated with severe acute respiratory infections in paediatric patients from 2016 to 2023 in Hangzhou, China

**DOI:** 10.1099/mgen.0.001484

**Published:** 2025-08-22

**Authors:** Yue Yu, Xinfen Yu, Feifei Cao, Xueling Zheng, Lijiao Ao, Shi Cheng, Xiaofeng Qiu, Yinyan Zhou, Jun Li

**Affiliations:** 1 Hangzhou Center for Disease Control and Prevention (Hangzhou Health Supervision Institution), 568 Mingshi Road, Shangcheng District, Hangzhou, Zhejiang, PR China; 2 Zhejiang Key Laboratory of Multi-Omics in Infection and Immunity, 568 Mingshi Road, Shangcheng District, Hangzhou, Zhejiang, PR China

**Keywords:** genomic surveillance, human respiratory syncytial virus, monoclonal antibody (mAb) resistance, paediatric, severe acute respiratory infection (SARI), sentinel surveillance programme

## Abstract

Human respiratory syncytial virus (HRSV), a leading cause of paediatric respiratory infections, is a major contributor to global childhood morbidity. The primary objective of this study was to investigate the epidemiology and phylogenetic characterization of HRSV in paediatric severe acute respiratory infection (SARI) cases in Hangzhou. A total of 2,277 paediatric SARI samples were screened for HRSV. Epidemiological trends, including age-specific positivity rates, were analysed. Whole-genome sequencing was performed on 113 HRSV-positive samples (subgroup A *n*=60, subgroup B *n*=53) to characterize genetic evolution. Phylogenetic analysis identified viral lineages and evolutionary rates. *In silico* binding free energy calculations assessed the impact of antigenic site mutations on monoclonal antibody (mAb) binding. In this study, disruptions in transmission patterns and age-specific positivity rates were observed in 2021 and 2023, coinciding with strict COVID-19 non-pharmaceutical interventions. A novel A.D.3.X lineage, circulating during 2021–2023 and defined by five unique aa substitutions in the G gene, was identified. The emergence of this lineage may have contributed to the observed epidemiological disruptions. *In silico* binding free energy calculations predicted that antigenic site mutations (N276S and I206M) in local strains reduce binding affinities for mAbs motavizumab and nirsevimab. These results emphasize HRSV’s genetic diversification in Eastern China and underscore the necessity for continuous genomic surveillance to track antigenic evolution and refine prevention strategies for high-risk paediatric populations.

**Impact Statement**

This study elucidates the evolving epidemiology and genetic diversity of human respiratory syncytial virus (HRSV) in Hangzhou, Eastern China, between 2016 and 2023. By integrating genomic surveillance with clinical datasets, we identified a novel HRSV-A lineage (A.D.3.X) and revealed dynamic shifts in subtype dominance, partially driven by COVID-19 non-pharmaceutical interventions. Crucially, we detected mutations in antigenic sites of the HRSV fusion protein, including residues associated with reduced binding affinities of monoclonal antibodies such as nirsevimab and motavizumab, raising concerns about potential therapeutic resistance. A revised phylogenetic nomenclature system was applied to enhance global strain classification, enabling precise tracking of HRSV evolution. These findings underscore the necessity of integrating real-time genomic surveillance into regional public health strategies to guide vaccine design, optimize prophylactic interventions and improve outbreak preparedness. This multidisciplinary approach, combining virological, genomic and epidemiological analyses, provides a model for addressing emerging viral threats, with direct implications for densely populated urban regions grappling with similar challenges globally.

## Data Summary

The 113 viral sequences analysed in this study are available in GenBank under accession numbers PV367284–PV367343 (subgroup A) and PV367344–PV367396 (subgroup B). GISAID accession numbers, reference sequences used and other supplement materials in this study are available online at https://doi.org/10.5281/zenodo.15632751. The Python script used in this study is available at https://github.com/Ice-YY/supplymental-Script.

## Introduction

Human respiratory syncytial virus (HRSV) is a single-stranded, negative-sense RNA virus belonging to the *Pneumoviridae* family [1]. HRSV is a leading cause of lower respiratory tract infections in young children globally, accounting for ~26% of hospital-attributable deaths among children aged 0–60 months with severe respiratory illness. In 2019 alone, HRSV-associated mortality reached an estimated 101,400 cases worldwide [2]. High-risk adult populations, including individuals with chronic cardiovascular diseases or immunocompetent adults aged over 65 years, also experience a disease burden comparable to that of seasonal influenza A [3]. Humoral immunity against HRSV is incomplete, permitting multiple recurrent infections throughout an individual’s lifetime [4]. Given this significant public health burden, the development of effective HRSV interventions has become a priority for pharmaceutical companies and research institutions. Currently, more than 30 candidate prophylactics and therapeutics employing diverse strategies, including vaccines, monoclonal antibodies (mAbs) and small molecule inhibitors, are undergoing clinical development [5]. Current prophylactic strategies against HRSV infection primarily employ mAbs targeting the fusion protein [6,8]. Palivizumab, the first licensed mAb targeting antigenic site II of the F protein, and its derivative motavizumab have demonstrated efficacy in reducing hospitalization rates and severe respiratory complications in high-risk paediatric populations and immunocompromised elderly patients [8,11]. However, the requirement for monthly intramuscular administration during HRSV season creates substantial cost barriers, particularly for low-income populations in resource-limited settings [6]. Recent advances in immunoprophylaxis include nirsevimab, a long-acting mAb targeting the antigenic θ site of the F protein. This agent enables single-dose protection throughout the HRSV season [6,12]. For adult populations, the RSVpreF3 recombinant subunit vaccine containing stabilized prefusion F protein has received regulatory approval in countries like the UK and the USA. This vaccine induces robust neutralizing antibody responses, demonstrating effectiveness against RSV-associated lower respiratory tract disease in older adults and pregnant people. The antibodies can also provide passive protection for infants in the first few months after birth [13].

The HRSV genome is ~15.2 kb in length and encodes 11 proteins. Among these, the two surface glycoproteins, the attachment glycoprotein (G protein) and fusion protein (F protein), play critical roles in viral entry and modulating host immune responses. HRSV is divided into two antigenic subgroups, A and B [14]. Historically, HRSV phylogenetic lineages were defined using phylogenetic analyses of the second hypervariable region within the viral G gene [15]. However, existing nomenclature systems for these lineages remain inconsistent. Over 10 HRSV-A and 30 HRSV-B lineages have been reported in the literature [16,18], with nomenclature inconsistencies arising from mixed criteria: some lineages are gene-based (e.g. GA1 and GB1), while others derive from geographic origins [e.g. THB (Thailand) [19], ON1 (Ontario) [20] and NA1 (Niigata) [21]]. This lack of a unified framework leads to ambiguity in lineage classification. Furthermore, the resolution of recently emerging lineages remains limited, as current classification systems for HRSV-A primarily rely on G gene sequence duplication to assign strains to the ON1 (HRSV-A). This approach may oversimplify genetic diversity, potentially obscuring finer-scale evolutionary patterns.

To address this problem, Goya *et al*. [22] proposed a revised nomenclature system in 2024. In this study, we applied the revised system, which not only improves phylogenetic resolution compared with previous methods but also enables lineage classification using full-length genome sequences. This approach enhances the accuracy and resolution of HRSV strain surveillance, offering a more robust framework for tracking viral evolution.

This study aims to conduct a comprehensive epidemiological and phylogenetic analysis of HRSV circulating in Hangzhou, a major metropolitan hub and demographically representative region of Eastern China between 2016 and 2023. These cases are associated with severe acute respiratory infection (SARI), as defined by the WHO criteria (acute onset of fever ≥38 °C, cough and hospitalization within 10 days of symptom onset). By integrating genomic surveillance with clinical data, this multidisciplinary study seeks to elucidate temporal trends in local HRSV transmission and monitor genetic variations within fusion genes to evaluate the protective efficacy of existing mAb therapies. The study will provide critical insights into the evolutionary dynamics of HRSV, informing regional strategies for prophylactic intervention, particularly for high-risk paediatric populations in densely populated urban centres.

## Methods

### Sample collection

This retrospective study used clinical specimens and associated patient data from the sentinel surveillance programme (SARI) conducted in Hangzhou between 2016 and 2023. Specimens included nasopharyngeal and oropharyngeal swabs. The specimens were collected and sent to the Hangzhou Center for Disease Control and Prevention’s lab to be tested within 24 h. Viral RNA was analysed using a commercial HRSV Real-Time PCR Diagnostic Kit (XABT, Beijing, China). Among the 2,277 SARI cases screened, 278 were confirmed positive for HRSV. De-identified patient data were used for epidemiological analysis.

### Virus detection

Samples with confirmed HRSV-positive results in initial records were selected for further analysis. Viral RNA was extracted from these specimens using Viral DNA and RNA Extraction Kit (T324H) (Tianlong, Xi’an, China) on a GeneRotex 96 automated extractor (Tianlong). A subsequent real-time PCR amplification assay was performed using a QuantStudio 5 thermocycler (Applied Biosystems, USA) with the 24-pathogen Multiplex Real-Time PCR Diagnostic Kit (XABT). In addition to HRSV, this diagnostic kit also screens for viral pathogens including influenza A, influenza B, human metapneumovirus (HMPV), human adenovirus, human rhinovirus, human parainfluenza virus (HPIV), enterovirus (EV) and human coronavirus (HCoV).

Samples exhibiting a Ct value ≤30 for HRSV from initial screening were subjected to subgroup determination via an additional real-time PCR assay using the HRSV A/B Diagnostic Kit (XABT). Based on successful subgroup determination and Ct ≤30, a subset of 131 samples was selected for whole-genome sequencing.

### Viral genome amplification and sequencing

Viral genome amplification was performed using an HRSV A/B amplicon-based sequencing kit (XABT). For cDNA synthesis, 10 µl of viral RNA was reverse-transcribed. PCR amplification was conducted under the following thermocycling conditions: initial denaturation at 98 °C for 30 s, 30 cycles of denaturation at 98 °C for 10 s, annealing at 58 °C for 30 s and extension at 68 °C for 2 min, using a Mastercycler® X50 thermocycler (Eppendorf, Germany).

PCR products were purified with 0.6X AMPure XP beads (Beckman Coulter, USA). Purified DNA was quantified using the 1X dsDNA HS Assay Kit on a Qubit 4.0 Fluorometer (Thermo Fisher Scientific, USA). Libraries were prepared from 100 ng of purified PCR products using the MagicPrep™ NGS system and the Revelo DNA-Seq Enz Library Preparation Kit (Tecan, Switzerland). Sequencing was performed on an Illumina MiSeq platform (Illumina, USA) with the MiSeq Reagent Kit v2 (300 cycles).

### Genome assembly

Raw sequencing reads were processed using fastp (v0.23.4) [23] to trim adapters and filter low-quality reads with default parameters. Primer sequences were removed using iVar (v1.4.3) [24] to ensure accurate alignment. Cleaned reads were mapped to the HRSV reference genomes [NC_038235.1 (subgroup A) and NC_001781.1 (subgroup B)] using BWA-MEM (r1243) [25] with default settings. SNP calling was performed using Clair3 (v1.0.10) [26]with the Illumina-specific model (20210517), followed by automatically determined filter thresholds. To ensure data integrity, sequences with a depth of coverage <5× across coding ORFs were excluded using SAMtools (v1.20) [27]. Consensus sequences were generated from Clair3-derived SNP files using BCFtools (v1.21) [27] and validated for frameshift errors via the Nextclade CLI (v3.10.2) [28]. One hundred thirteen genomes were retained for phylogenetic and evolutionary analyses in this study.

### Phylogenetic analysis

Sequence processing, including format conversion, sequence extraction, translation, annotation and visualization, was performed using Geneious Prime (v2025.0). To resolve newly designated lineages, phylogenetic tree construction followed the methodology of Goya *et al*. [22]. Phylogenetic trees were reconstructed using IQ-TREE2 (v2.3.6) [29] with 10,000 ultrafast bootstrap replicates and 1,000 Shimodaira–Hasegawa approximate likelihood ratio test (SH-aLRT) replicates to assess nodal support. A clade was considered monophyletic if it had an SH-aLRT value ≥80% and an ultrafast bootstrap value ≥90%. The substitution model was automatically selected by the ModelFinder module based on the lowest Bayesian information criterion score [29] [30]. Representative sequences (160 for HRSV-A and 89 for HRSV-B) from designated lineages by Neher *et al*. [31] were included to refine lineage classification. Final trees were annotated and visualized using iTOL [32].

To compare the genetic lineages of local strains with global trends, a dataset of HRSV group A and group B sequences collected globally between 2016 and 2023 was analysed. These sequences were sourced from the GISAID database [33]. Quality filtering was performed for all sequences using the Nextclade CLI. Sequences classified as ‘mediocre’ or ‘bad’ based on predefined quality control metrics were excluded from downstream analyses.

To estimate the evolutionary rates of the coding sequence (CDS), local sequences were aligned to reference genome CDSs, and phylogenetic reconstructions were performed as described previously. Evolutionary rates were calculated using TreeTime (v0.11.4) [34] under the molecular clock model.

### Antigenic site analysis

Antigenic sites θ and I–V [35,36] were defined based on a pairwise distance threshold of 5 Å from the following crystal structures: site θ (PDB: 4JHW [37] and 5W23 [38]), site I (PDB: 6ABP [39]), site II (PDB: 4ZYP [40]), site III (PDB: 5U68 [41]) and site IV (PDB: 3O45 [42]). Viral sequences in this study were aligned to ATCC strains HRSV A subgroup (KT992094) and HRSV B subgroup (AY353550) to ensure compatibility with the sequences corresponding to the crystal structures. Mutations within sites θ and II, which are relevant to the binding of motavizumab and nisevimab (mAb currently used for HRSV prevention), were selected for subsequent binding free energy (ddG) calculations.

### Computational binding affinity analysis

To perform *in silico* binding free energy calculations, the crystal structures of motavizumab (PDB: 4ZYP) and nisevimab bound to HRSV-A (PDB: 5UDC [43]) and HRSV-B (PDB: 5UDD [43]) were utilized. The Rosetta software suite (v3.14) and the Flex ddG protocol [44] were employed to estimate changes in mAb-antigen binding affinity. Antigen-binding fragments were extracted using the Rosetta clean pdb script. Structural visualization and sequence analysis were conducted using ChimeraX (v1.9).

For ddG calculations, each aa substitution at sites θ and II was evaluated with the following parameters: maximum minimization iterations=5,000; absolute score convergence threshold=1; number of backrub trials=35,000; and repeats (nstruc)=50. The F protein chain was designated as the moving chain during simulations. Mutations were input into Rosetta using its standardized format.

### Statistical analysis

Data consolidation was performed using Biopython packages (v1.26.4) [45] and custom Python scripts. Chi-square tests were employed to assess significant differences between categorical variables using R (v4.4.2) [46] and RStudio (2024.12.0 Build 467). Data wrangling, temporal analysis and visualization were performed with the following R packages (compatible with v4.4.2): tidyverse, lubridate, ggplot2, seqinr and GraphPad Prism (v10.1.2). Statistical significance was defined as *P*≤0.05. Regional monthly average maximum and minimum temperatures were calculated using data derived from the W3S platform [47].

## Result

### Epidemiology of HRSV in Hangzhou (2016–2023)

**Fig. 1.**
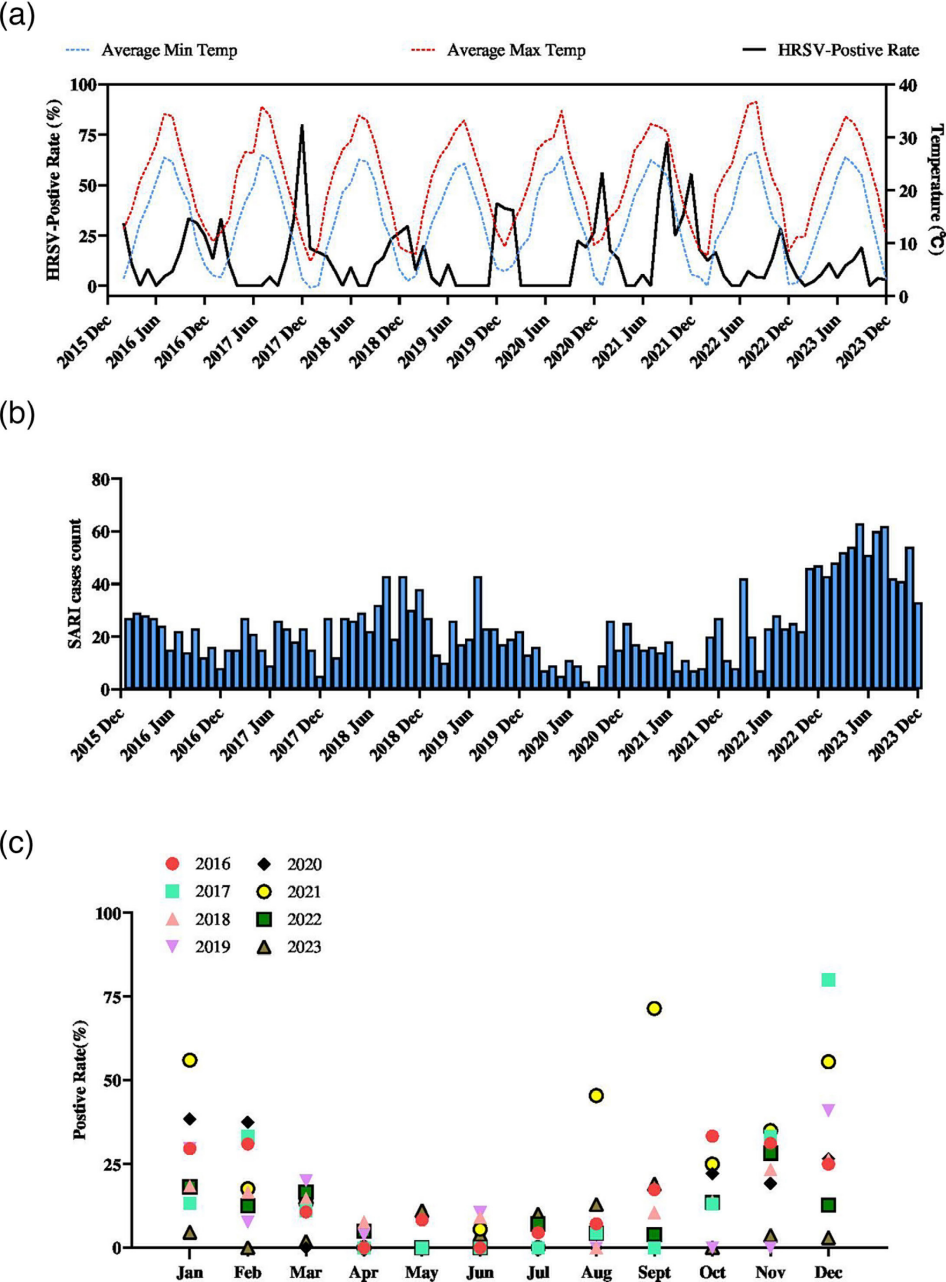
SARI case seasonal trends and HRSV-positive rate. (**a**) The monthly positive rate of HRSV among SARI cases correlated with average monthly minimum and maximum temperatures, with most epidemic waves aligning with seasonal trends. An atypical outbreak was observed during the summer of 2021. (**b**) Monthly SARI case counts from 2016 to 2023 are shown, revealing a 1-month gap in September 2020. This absence likely reflects stringent non-pharmaceutical interventions that suppressed viral transmission, resulting in no cases meeting the SARI case definition during that month. (**c**) Comparison of monthly positivity rates across the 8-year period revealed unusual summer outbreaks in August and September 2021, as well as a modestly elevated positivity rate in August 2023.

Among the 2,277 SARI cases screened through the surveillance programme during 2016 to 2023 (Fig. 1a, b), 278 (12.2%) tested positive for HRSV. A significant year-to-year variation in prevalence was observed: 2021 exhibited the highest positivity rate (29.3%, 54/184), while 2023 showed the lowest (6.6%, 40/603). Age-stratified analysis revealed significant differences across groups. The highest prevalence occurred in infants aged 0–12 months (20.7%, 50/242), whereas the lowest was observed in children aged 6–11 years (2.7%, 18/672). In 2021, the prevalence among children aged 2–5 years (27.6%, 27/98) was significantly higher than the overall average (13.9%, 137/984) (Table 1). Co-infection with other viral pathogens occurred in 58 of 278 cases (20.9%), including 5 cases involving 3 distinct viruses. The identified pathogens were rhinovirus (*n*=15), adenovirus (*n*=10), HPIV (*n*=9), bocavirus (*n*=8), HMPV (*n*=7), influenza A (*n*=6), EV (*n*=4), influenza B (*n*=3) and HCoV (*n*=1). Additionally, three cases were co-infected with both HRSV-A and HRSV-B. Seasonal trends demonstrated shifts in peak HRSV-positive rate during the study period. In both 2021 and 2023, biannual peaks were observed, diverging from the typical winter predominance to include summer surges (Fig. 1c).

**Table 1.** HRSV positivity rates among SARI cases: annual trends and age-stratified case profiles (2016–2023)

Year	SARI case	HRSV-positive case (%)	Age-specific distribution									
			0–12 months		13 months−2 years		2–5 years		6–11 years		12–18 years	
			*n*	Pos *n* (%)	*n*	Pos *n* (%)	*n*	Pos *n* (%)	*n*	Pos *n* (%)	*n*	Pos *n* (%)
2016	245	39 (15.9)	47	15 (31.9)	54	10 (18.5)	99	11 (11.1)	43	2 (4.6)	2	1 (50)
2017	212	23 (10.9)	35	6 (17.1)	44	6 (13.6)	101	11 (10.8)	31	0 (0)	1	0 (0)
2018	348	40 (11.5)	55	5 (9.1)	62	10 (16.1)	150	20 (13.3)	76	5 (6.6)	5	0 (0)
2019	259	23 (8.9)	21	6 (28.6)	45	7 (15.6)	116	9 (7.8)	73	1 (1.4)	4	0 (0)
2020	123	22 (17.9)	22	5 (22.7)	25	7 (28)	56	8 (14.3)	19	0 (0)	1	0 (0)
2021	184	54 (29.3)*	27	8 (29.6)	40	18 (45)	98	27 (27.6) *	16	0 (0)	3	1 (33.3)
2022	303	37 (12.2)	17	2 (11.8)	26	2 (7.7)	151	29 (19.2)	105	4 (3.8)	4	0 (0)
2023	603	40 (6.6)*	18	3 (16.7)	43	8 (18.6)	213	22 (10.3)	309	6 (1.9)	20	1 (5)
Total	2,277	278 (12.2)	242	50 (20.7)	339	68 (20.1)	984	137 (13.9)	672	18 (2.7)	40	3 (7.5)

Age group classified by NICHD paediatric age groups and year.

*Statistically significant differences, as determined by chi-square test *P*-values adjusted for multiple comparisons using the false discovery rate (FDR) method. The null hypothesis states no significant difference in HRSV positivity rates between the specified group and other groups.

### Phylogenetic diversity and lineage classification

**Fig. 2.**
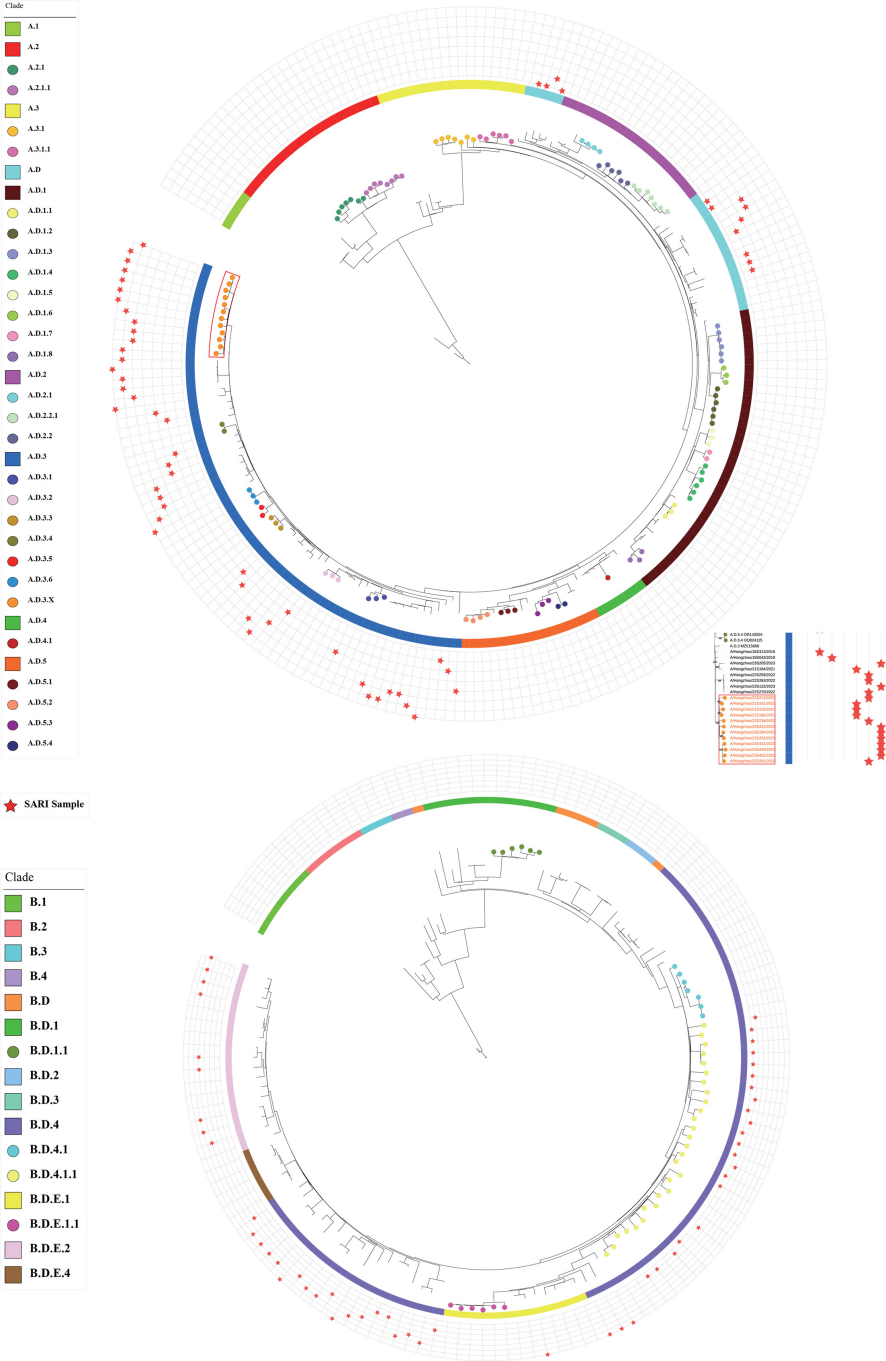
Phylogenetic trees of HRSV-A and HRSV-B. Maximum-likelihood phylogenetic trees of HRSV-A (top) and B (bottom) between 2016 and 2023, with lineages highlighted by a combination with coloured stripes adjacent to corresponding clades and coloured dot representing the sub-clade. Nodes marked with red-filled stars denote locally sequenced strains from Hangzhou, China, and their sampling year. The novel clade A.D.3.X is highlighted with a red frame. Next to the HRSV-A’s phylogenetic trees is a zoom-in figure for the novel clade; the bootstrap values are 100/100 for this branch.

Whole-genome sequencing was performed on 113 HRSV genomes (60 HRSV subgroup A and 53 HRSV subgroup B) to construct maximum-likelihood phylogenetic trees (Fig. 2). A distinct cluster of 12 highlighted in orange represents a novel putative lineage A.D.3.X within the A.D.3 lineage, circulating between 2021 and 2023. This novel lineage meets the criteria proposed by Goya *et al*. [22] and is characterized by five unique aa substitutions in the G protein (V225A, Y273H, L274P, L298P and L310P) compared with the parental A.D.3 lineage.

**Fig. 3.**
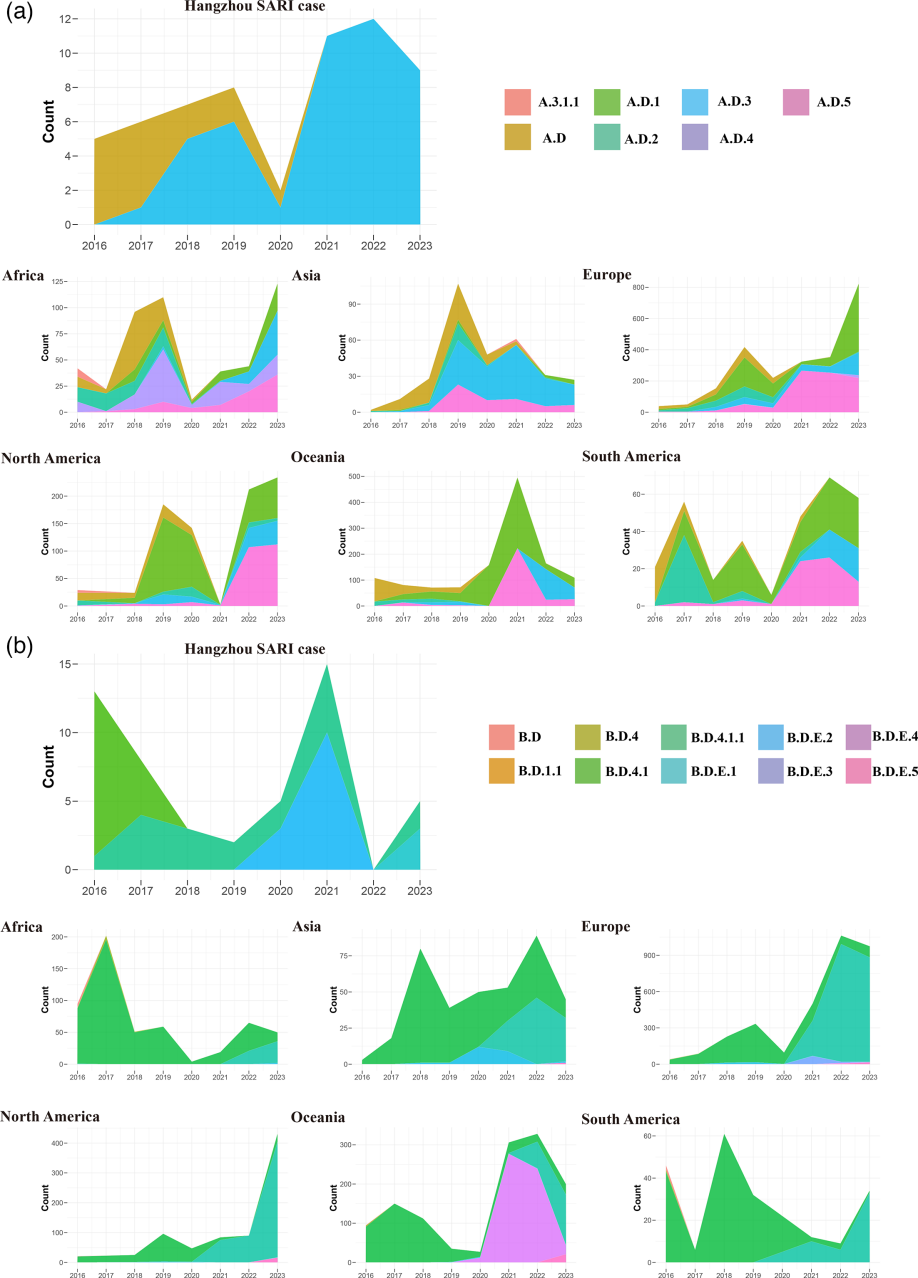
Lineage composition of HRSV-A and HRSV-B locally and globally. (**a**) Local and global (stratified continentally) lineage composition of HRSV-A strains (2016–2023), presented as stacked area plots, revealed a shift in dominant lineages during the study period. (**b**) Similarly, local and global (stratified continentally) lineage composition of HRSV-B strains (2016–2023), visualized as stacked area plots, showed less pronounced lineage shifts in some continents compared with HRSV-A.

Global analysis of circulating lineages from the GISAID datasets revealed distinct lineage distributions across six continents. Most regions experienced lineage shifts during the COVID-19 pandemic period for both subgroups A and B (Fig. 3a, b).

Temporal analysis of local circulating lineages demonstrated dynamic shifts in the predominance of HRSV-A and HRSV-B subtypes (Fig. 3a, b). For HRSV-A, lineage A.D dominated during 2016–2017, while A.D.3 emerged in 2017 and became predominant from 2018 onwards. Among HRSV-B, lineage B.D.4.1 predominated from 2016 to 2019, followed by B.D.E.2 dominance during 2020–2021. In 2023, co-circulation of B.D.E.1 and B.D.E.2 was observed. Notably, no HRSV-B subgroup cases were detected in 2022.

### Entropy and evolutionary rate analysis

**Fig. 4.**
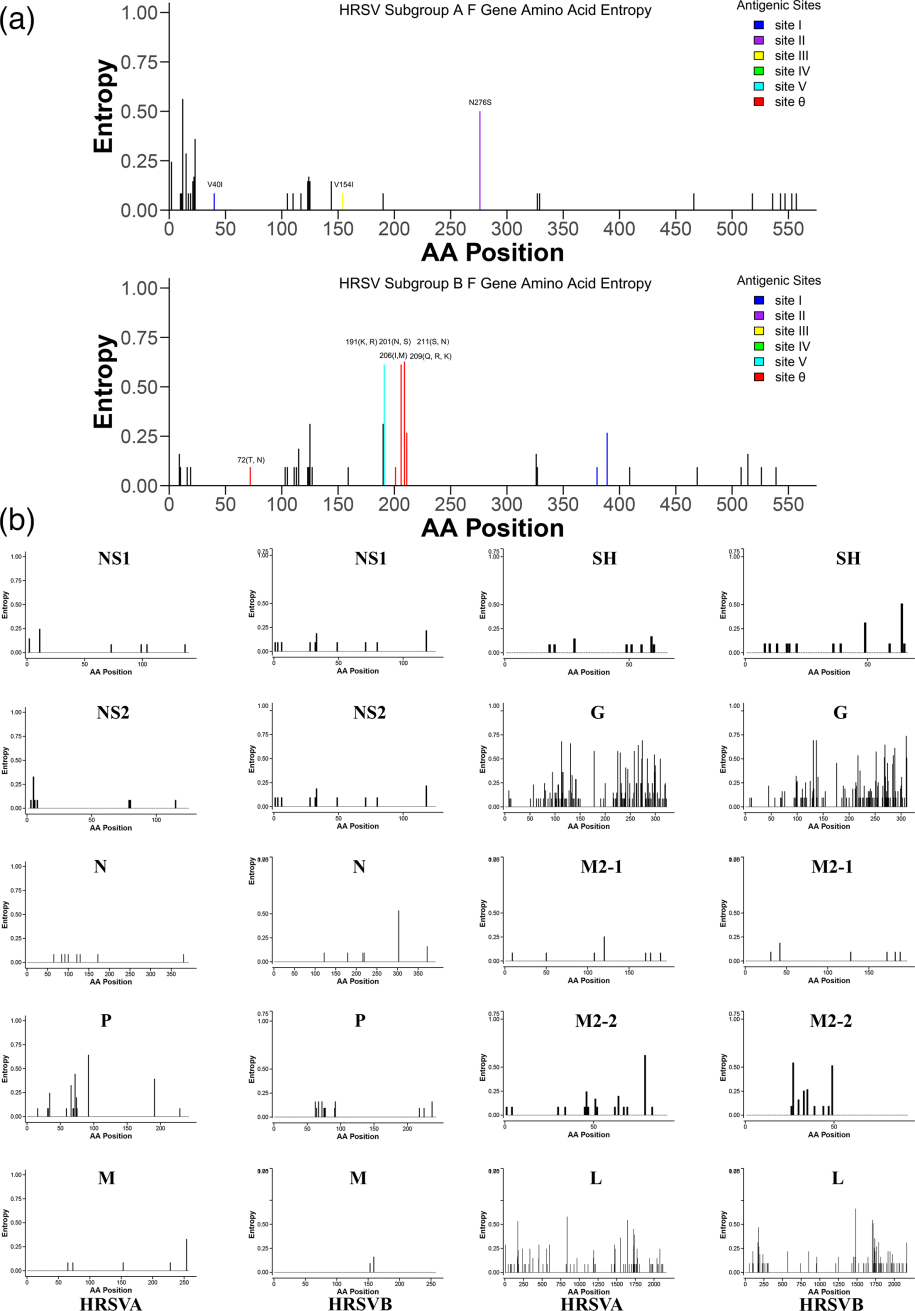
Entropy analysis for genes of HRSV-A and HRSV-B. (**a**) Entropy analysis of the F gene in HRSV subgroups A and B revealed aa substitutions at antigenic sites, which were colour-highlighted in the graphical representation. Polymorphism at these sites was annotated directly on the figure. (**b**) Entropy analysis of the other genes, highlighting divergent positions with distinct diversity patterns between subgroups A and B.

**Fig. 5.**
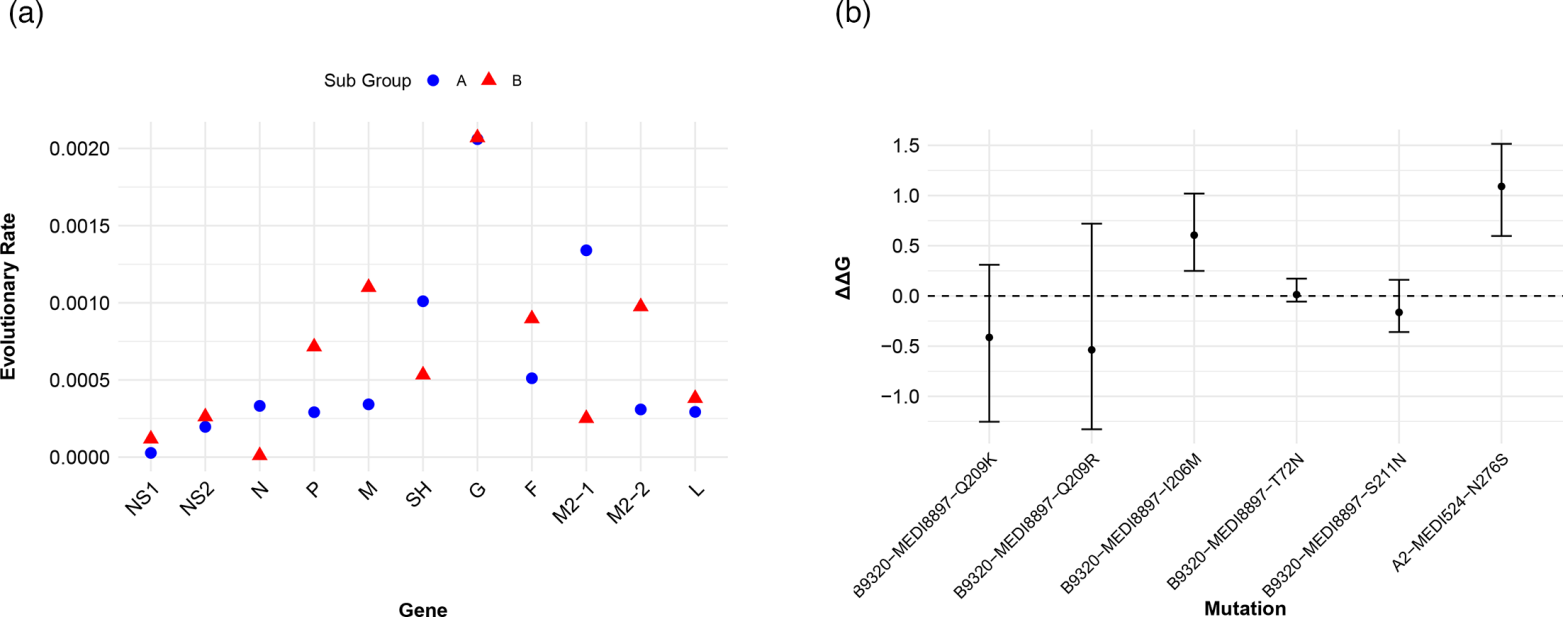
Evolutionary rate and docking simulation for HRSV-A and HRSV-B. (**a**) Evolutionary rate comparisons between HRSV subgroups A and B. (**b**) Rosetta flex ddG results for mutations. The black dot indicates the average ddG in 50 structures simulated, and an error bar indicates the range of the calculated ddG. A negative value refers to increased stability of antigen-antibody complexes, and positive values refer to decreased stability.

The analysis of local strains in Hangzhou (2016–2023) revealed distinct aa entropy patterns between the two subgroups (Fig. 4a, b). The G gene exhibited the highest evolutionary rate in both subgroups, while the evolutionary rate of the F gene was estimated at 5.10×10^−4^ substitutions/site/year for subgroup A, compared with 8.97×10^−4^ substitutions/site/year for HRSV-B, indicating a higher substitution rate in the latter. Additionally, the NS1, NS2, N and L genes were more conserved compared with the P, M and M2 genes (Fig. 5a).

### Antigenic site mutations

Multiple aa substitutions were identified in the fusion gene of both HRSV-A and HRSV-B strains. Comparative analysis revealed lower conservation across antigenic sites in HRSV-B compared with HRSV-A in this study.

To evaluate potential impacts on mAb efficacy, substitutions of interest [T72N, I206M, Q209K, Q209R and S211N (HRSV-B; bind nirsevimab) and N276S (HRSV-A; bind motavizumab)] were analysed using Rosetta-based flex-ddG. Computational modelling indicated that N276S and I206M have ddG>0 across all 50 simulated structures, suggesting that these mutations significantly impair the stability of antigen-antibody complexes. This computational evidence indicates that N276S and I206M may confer potential resistance mechanisms against motavizumab and nirsevimab, respectively (Fig. 5b).

## Discussion

This comprehensive 8-year study highlights critical trends in HRSV infections among paediatric SARI cases in Hangzhou, a major metropolitan area with a population exceeding 10 million and one of China’s largest transportation hubs. Our data demonstrate substantial disease burden from HRSV-associated morbidity, with highest prevalence rates observed in infants aged 0–24 months, consistent with global infection patterns [2,8, 48]. Surveillance identified a notable epidemiological anomaly: HRSV-positive SARI cases surged unexpectedly in 2021, followed by a marked decline in 2023. These incidence fluctuations coincided with atypical biennial peak shifts during the post-pandemic period, suggesting substantial epidemiological disruption from COVID-19 non-pharmaceutical interventions [7]. Of particular clinical significance, children aged 2–5 years exhibited disproportionately high HRSV positivity rates in 2021, potentially reflecting immunological vulnerability from extended school closures and online education mandates during peak transmission seasons. These findings align with a report from the U.S. Military Healthcare System documenting similar age-specific case surges and shifts in outbreak peak during 2022 [48]. As this study focused on SARI cases, the findings reflect not only the positivity rate among paediatric populations but also highlight the clinical and societal burden of HRSV infections, which disproportionately affect vulnerable individuals requiring hospitalization and may lead to severe consequences. Specifically, we advocate for expanded prophylactic measures to protect high-risk paediatric populations during anticipated resurgent outbreaks.

Beyond temporal trends, shifts in circulating HRSV lineages may critically influence epidemic dynamics, with pandemics potentially accelerating such transitions. Epidemiological surveillance in Ireland utilizing the updated phylogenetic nomenclature system revealed significant HRSV lineage shifts before and after the COVID-19 pandemic. Additionally, a novel descendant lineage originating from A.D.1 was identified during this period [49]. Local genomic surveillance also revealed distinct subtype dominance patterns. While HRSV-A lineages transitioned from A.D to A.D.3 in 2018, HRSV-B exhibited intermittent circulation of dominant lineages, with no cases detected in 2022. In contrast, a Korean study analysing pre- and post-pandemic HRSV sequences using traditional lineage classification reported no significant lineage changes [50]. This discrepancy may stem from differences in phylogenetic analytical frameworks, as the enhanced resolution of the updated nomenclature system could allow for more nuanced tracking of viral lineage dynamics. These observations highlight the potential advantages of the refined classification approach in supporting precision. Notably, a novel HRSV-A lineage (A.D.3.X) emerged in 2021 and became one of the predominant lineages from 2021 to 2023. The sustained prevalence of HRSV-A during HRSV-B’s decline (2022–2023) may indicate adaptive advantages that facilitated its transmission during the pandemic, highlighting the importance of continuous genomic surveillance. Proactive monitoring of viral evolutionary trajectories remains critical for anticipating resurgences and designing region-specific interventions.

MAbs significantly reduce HRSV-related hospitalization rates, representing a pivotal advancement in prophylaxis [6,9, 12]. In China, nirsevimab was approved in 2024 and widely available for susceptible populations. However, the efficacy of these therapies depends on the stability of viral epitopes they target [51,53]; thus, continuous monitoring of HRSV genetic variation, particularly focusing on the F protein, is critical to mitigate immune escape risks and sustain long-term intervention success. Studies have pointed out that the clinical isolates carrying mutations such as N262D, K272E/M/Q and S275F/L exhibit reduced susceptibility to palivizumab neutralization [54,56]. The N276S substitution remains controversial [51,54], as evidence suggests that it may confer resistance to mAbs, potentially classifying it as a mAb-resistant mutant [52,57]. Another study employed recombinant HRSV-A and HRSV-B strains to evaluate nirsevimab resistance, with subsequent analysis of binding site conservation and its impact on neutralization potency. Notably, K68E in HRSV-A and K68N, N201S and N201T in HRSV-B caused nisevimab neutralization escape. The I206M mutation caused a 5.0-fold reduction in neutralization activity against HRSV-B; however, this mutation frequently co-occurs with Q209R, which restores and enhances nirsevimab potency against HRSV-B [53]. These findings were consistent with our *in silico* predictions, in which I206M and N276S were predicted to reduce susceptibility to mAbs, whereas Q209R enhanced the stability of antigen-antibody complexes. This underscores the necessity of continuous surveillance for aa substitutions in antigenic sites and their potential impact on mAb neutralization. Additionally, less conservation of antigenic sites in HRSV-B compared with HRSV-A [16] highlights its greater potential for immune evasion and resistance to mAb, emphasizing the importance of subtype-specific monitoring.

## Study limitations and conclusion

This study may reflect sampling biases inherent to only including SARI cases. Additionally, the samples used in this study have been preserved for a long time, which precluded *in vitro* validation of the impacts of antigenic mutations in this study.

Despite these limitations, our findings emphasize the urgency of integrating genomic surveillance with clinical data to optimize regional prevention strategies.

## Abbreviations

CDS: coding sequence
EV: enterovirus
HCoV: human coronavirus
HMPV: human metapneumovirus
HPIV: human parainfluenza virus
HRSV: human respiratory syncytial virus
mAb: monoclonal antibody
SARI: severe acute respiratory infection
SH-aLRT: Shimodaira–Hasegawa approximate likelihood ratio test
